# A Deep Learning Method for the Prediction of the Index Mechanical Properties and Strength Parameters of Marlstone

**DOI:** 10.3390/ma15196899

**Published:** 2022-10-05

**Authors:** Mohammad Azarafza, Masoud Hajialilue Bonab, Reza Derakhshani

**Affiliations:** 1 Department of Civil Engineering, University of Tabriz, Tabriz 5166616471, Iran; 2 Department of Earth Sciences, Utrecht University, 3584 CB Utrecht, The Netherlands

**Keywords:** geomechanical properties, deep learning, rock material, rock strength parameters, marlstone

## Abstract

The index mechanical properties, strength, and stiffness parameters of rock materials (i.e., uniaxial compressive strength, c, ϕ, E, and G) are critical factors in the proper geotechnical design of rock structures. Direct procedures such as field surveys, sampling, and testing are used to estimate these properties, and are time-consuming and costly. Indirect methods have gained popularity in recent years due to their time-saving and highly accurate results, which are comparable to those obtained through direct approaches. This study presents a procedure for establishing a deep learning-based predictive model (DNN) for obtaining the geomechanical characteristics of marlstone samples that have been recovered from the South Pars region of southwest Iran. The model was implemented on a dataset resulting from the execution of numerous geotechnical tests and the evaluation of the geotechnical parameters of a total of 120 samples. The applied model was verified by using benchmark learning classifiers (e.g., Support Vector Machine, Logistic Regression, Gaussian Naïve Bayes, Multilayer Perceptron, Bernoulli Naïve Bayes, and Decision Tree), Loss Function, MAE, MSE, RMSE, and R-square. According to the results, the proposed DNN-based model led to the highest accuracy (0.95), precision (0.97), and the lowest error rate (MAE = 0.13, MSE = 0.11, and RMSE = 0.17). Moreover, in terms of R^2^, the model was able to accurately predict the geotechnical indices (0.933 for UCS, 0.925 for E, 0.941 for G, 0.954 for c, and 0.921 for φ).

## 1. Introduction

Geotechnical design is usually faced with a great number of geomechanical uncertainties which require the conducting of various procedures to provide proper information as input data. This information is mainly provided with instrumentations, measurements, geotechnical testing, pilot boreholes, drilling, excavations, and field surveys [1,2], with the main goal of providing the geomechanical properties and stiffness parameters (i.e., deformational characteristics, pore pressure, and geomechanical properties) of rock materials [3,4,5]. In this regard, different procedures are performed to estimate these geomechanical indices, which can be classified as direct and indirect approaches [6,7]. Direct measurement techniques are the experimental methods that are applied to the rock mass in the field or to the rock material in the laboratory. To perform these experiments, there are usually special instructions, devices, and methods that are determined during different stages [8]. These methods are standardized by global organizations such as the American Society for Testing and Materials (ASTM), the International Society for Rock Mechanics (ISRM), and the British Standards Institution (BSI). Although the role of estimating geomechanical properties for rock masses guarantees the success of design and the assurance of construction, estimating these features in the field by using direct procedures is very costly and time-consuming. Thus, the application of alternative methods which can provide useful information with less cost and time is always welcome. Indirect techniques, which have received significant attention worldwide, can provide reliable information on the geomechanical characteristics of rock masses. In general, indirect techniques mostly use empirical and computer-based methods. These methods can be classified as probabilistic or geostatistical methods ranging from simple and multiple regression techniques to complicated relations which are established to provide an empirical relationship between the geomechanical indices [9]. Computer-based methods are mainly developed to predict parameters by using overlapping or the interpolation of the input data. In recent years, these trends have undergone significant changes [10]. Machine learning procedures such as artificial neural networks [11], fuzzy logic [12], genetic-fuzzy [13], soft-computing [14], adaptive neuro-fuzzy inference [15], support-vector machines [16], and multilayer perceptron [17] have been utilized to develop predictive models and to determine the required geomechanical indices and rock material strength parameters [18]. On the other hand, the application of artificial neural networks has attained significant success in providing predictive models to estimate the geomechanical properties of rock masses [19].

Singh et al. [20] used multi-layered feed-forward neural networks to investigate the petrographic features of schistose rocks. The main focus in providing a predictive model for rocks entails the uniaxial compressive strength (UCS) variation in rock materials, which is verified by experimental test results and error models such as mean absolute percentage error (MAPE) as the performance measure. Sonmez et al. [21] utilized the multilayer perceptron to estimate the Elastic modulus (E) of the rock materials based on UCS and empirical geomechanical classifications such as the Rock Mass Rating, RMR [22] and the Geological Strength Index, GSI [23]. The authors used percentage error and Root Mean Square Error (RMSE) as a performance justifier. Yılmaz and Yuksek [24] provided a multilayer perceptron-based predictive model to investigate E, UCS, and point load strength (I_s_) on gypsum specimens that were recovered from Sivas, Turkey. In 2009, they used an adaptive neuro-fuzzy inference to evaluate the variation of the geomechanical properties of Sivas gypsum [25]. Kahraman et al. [26] applied a multilayer perceptron to estimate the strength and deformability properties of Misis Fault Breccia in Nigde, Turkey. The prepared model was compared with the regression models which indicated that the predictive model showed good performance in estimating the indices.

Dehghan et al. [27] applied multi-layered feed-forward neural networks and standard regression techniques for the assessment of the geomechanical indices of several travertine samples. Bahrami et al. [28] used multivariate regression analysis and multilayer perceptron for evaluating the UCS, E, and fragmentation index. They used RMSE for model verification. Yurdakul et al. [29] applied a multilayer perceptron to estimate the UCS, E, and Schmidt hardness variations on 37 different carbonate rocks (e.g., marble, limestone, and travertine). The criteria used to evaluate the predictive performances were Coefficient of Determination (R^2^), Variance Account For (VAF), and RMSE. Majdi and Rezaei [30] used multivariable regression and multilayer perceptron for preparing the predictive model of UCS, E, and Schmidt hardness on 93 different rock types (i.e., igneous, metamorphic, and sedimentary rocks). They used the R^2^, RMSE, VAF, Mean Absolute Error (MAE), and Mean Relative Error (MRE) to establish a comprehensive error model as a performance control.

Asadi et al. [31] utilized the optimal fuzzy model to predict the strength and stiffness parameters of rock materials. They developed the Multi-objective Genetic Algorithm (MOGA) to estimate the geo-material indices. Their results showed that MOGA was capable of achieving proper results that were comparable to the results obtained with other artificial intelligence techniques. Armaghani et al. [32] used a swarm optimization-artificial neural network-integrated model to investigate the rock material geomechanical indices and the strength properties of 230 shale samples recovered from various excavation sites in Malaysia. Armaghani et al. [33] performed multilayer perceptron to predict the geomechanical properties like UCS, E, I_s_, Schmidt hammer rebound (R_n_), and P wave velocity (V_P_) of granite samples recovered from the grounds of the Pahang-Selangor tunnel in Malaysia. The RMSE, R^2^, and VAF were used as verification controls on the results of the model.

Ferentinou and Fakir [34] utilized a multi-layer perceptron to prepare a predictive model for investigating the geomechanical properties of various sedimentary and igneous rocks recovered from eastern KwaZulu-Natal, South Africa. The UCS and E were the main targets of their analysis, which was checked by the MSE. Asadi [35] considered shallow learning and multilayer perceptron to provide a predictive model that was targeted to estimate the UCS and E of several rock specimens collected from a sandstone reservoir in Iran. Asadi used the MSE and R^2^ to justify the model. Abdi et al. [36] developed a forecasting model to obtain the relation between UCS and E in sedimentary rocks by using multivariable regression analysis and a multilayer perceptron network. Hassanvand et al. [37] used multiple linear regression, multilayer perceptron and radial basis function approaches to estimate and predict the UCS and some basic physiomechanical features of carbonate rock materials recovered from the Iranian oil reservoir. The researchers utilized RMSE and VAF error measurements in their data set.

Heidari et al. [5] applied both the shallow multiple linear regression and the fuzzy logic method to predict the UCS of several rock material types. Umrao et al. [38] used the ANFIS procedure to predict the UCS and E of various sedimentary rocks. Mahdiabadi and Khanlari [39] used multiple linear regression, multiple nonlinear regression, multilayer perceptron, and adaptive neuro-fuzzy inferences systems for UCS and E prediction. They used 80 samples that were recovered from calcareous mudstones of the Aghajari Formation located in Tehran, Iran, as the main data set. Haider Rizvi et al. [40] applied feed-forward multi-layer perceptron, Stochastic Gradient Descent (SGD), and Batch Gradient Descent (BGD) algorithms by the Adam optimizer to correlate the seismic wave velocities (VP) of metamorphic reservoir host rock specimens that were recovered from the surface down to a depth of 10 km. Barham et al. [7] conducted the ordinary multivariate regression analysis and perceptron neural network to prepare a predictive model of UCS and E for various masonry specimens in Jordan. Miah et al. [41], by using the Coupled Simulated Annealing (CSA) optimizer and MLP-SVM method, attempted to predict the UCS of wellbore logging data. Sun et al. [18] provided a comparative study using the Chi-square Automatic Interaction Detector (CHAID), Random Forest (RF), SVM, k-NN, and MLP to predict the rock brittleness index. The results of the study suggested that the RF method (R^2^ = 0.971) resulted in the highest performance. Kardani et al. [42] used two types of conventional neural network algorithms and a modified equilibrium optimizer to predict the permeability of carbonate rocks. The results indicated that the predictability of the applied model led to RMSE = 0.0612/MAE = 0.0442 and RMSE = 0.0806/MAE = 0.0660 error rates in the training and testing stages, respectively. Siddig et al. [43] utilized the ANFIS and MLP on extensive data logs recovered from a drilled well to predict the Poisson’s ratio variation on host sedimentary rocks. Both methods provided an R^2^, MLP, and ANFIS of 0.97, 0.98, and 0.97, respectively.

Although the studies have been reflected as invaluable and significant achievements, they are consequences of shallow learning approaches. Shallow learning, (such as multilayer perceptron, MLP) is a type of machine learning where the learning is from the data described by pre-defined features. In deep learning, since feature extraction is computed automatically without manual human intervention, this method has gained popularity. In addition, deep learning often outperforms conventional shallow learning methods since it extracts informative features automatically from raw data with little or no pre-processing due to its complex architecture [44]. He et al. [45] provided a fast and reliable estimation of rock material geomechanical properties by conducting a deep convolutional neural network (CNN) technique to predict rock material strength and stiffness parameters with high accuracy. The study presented herein provides a deep learning method by using the deep neural network procedure to prepare a predictive model to estimate the geomechanical properties, strength, and stiffness parameters of marlstones, which are considered to be one of the most complicated geo-materials.

## 2. Objective of Study

Geomechanical characteristics of rock materials are regarded as crucial factors for the safe design and effective implementation of geotechnical structures in host rocks. Such measurements require an extensive field investigation, sample preparation, and testing (depending on the scale of the site and the aim of the project). The preparation of relevant information such as strength and stiffness parameters about rock materials by using direct methods is time-consuming and expensive. The main costs of geomechanical investigation are related to the sampling, preparation, and geotechnical tests which are highly time-consuming in addition to requiring funding. Consequently, the use of indirect methods for determining the rock material’s properties can significantly reduce costs and time if they are reliable and accurate. The application of machine learning and deep neural networks has shown that they can be used as a complementary approach to predicting geomechanical properties. The work presented herein attempts to use a machine learning-based predictive model to predict the geotechnical features of marly rocks. The model has attempted to predict the strength and stiffness parameters of rock materials. The model may be considered to provide a complementary procedure to assess the geotechnical characteristics of the marly rock materials in the South Pars region, southwest Iran.

## 3. Geomechanical Indices of Rock Materials

### 3.1. Site Location and Geological Setting

**Figure 1:**
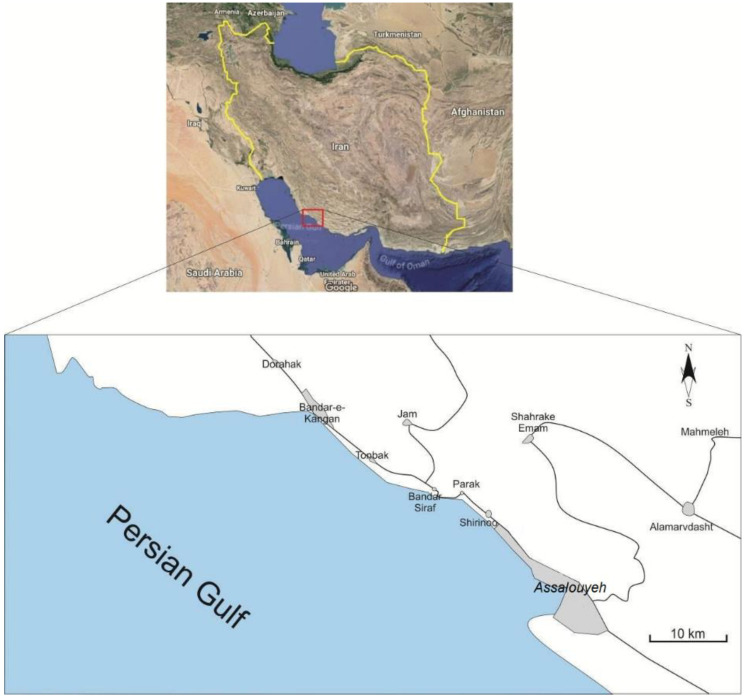
Location map of the South Pars region in Iran.

Marl is one of the most complicated geo-materials with a highly variable and complex geotechnical and geomechanical behavior that is classified as weak sedimentary rock or hard soil. Marlstone (rock form of marl) is a carbonate-clay complex that contains variable amounts of clay and silt. The presence of clay particles from almost all types of clay groups leads to a wide spectrum of the geotechnical behavior of marlstones. In addition, this behavior can further vary with the variation of the percentage of carbonate or clay content. Table 1 presents a classification of marls based on carbonate-clay content [46]. According to this table, marls may contain 25% to 75% carbonate. Marls are present in many parts of Iran, especially in southwest Iran. The South Pars region is one of the regions in southwest Iran with extensive marl outcrops that are subjected to various construction projects. Since these constructions have led to many geotechnical problems, comprehensive geomechanical studies have been conducted in this region. The study presented in this article provides an alternative procedure with high accuracy to estimate the geomechanical properties of marlstone in the South Pars region (Figure 1). The region geologically originates from the late Neo-Proterozoic (Hormuz series) to the Quaternary (recent alluviums). The Mishan, Aghajari, and Bakhtiari formations of the South Pars region extensively contain marl and marlstones.

### 3.2. Experimental Framework

Geomechanical and strength indices like UCS, cohesion (c), angle of internal friction (φ), elastic modulus (E), shear modulus (G), bulk modulus (K), and P-wave modulus (M) are considered to estimate the durability of rock materials/masses in geotechnical projects [26]. In this study, a total of 120 specimens were recovered from different spatial locations of the marly outcrops of the South Pars region, with particular care taken to cover the entire region. After sampling, the specimens were transferred to the laboratory to perform geomechanical tests such as uniaxial compressive strength and direct-shear tests in accordance with ISRM and ASTM. Uniaxial compressive strength (UCS) is the maximum axial compressive stress that a right-cylindrical sample of material can withstand before failing. It is also known as the unconfined compressive strength of material because the confining stress is set to zero. UCS represents the compressive strength of the rock, which can be defined as the ability of the rock to resist forces imposed on it or the maximum stress that a sample can withstand under specified loading conditions. ASTM D7012 [46] provides a description of the experimental procedure for the testing of samples. A direct shear test is a laboratory or field test used by geotechnical engineers to measure the shear strength properties of soil or rock material or discontinuities in soil or rock masses. ASTM D5607 [47] provides a detailed description of the test preparation and procedures. Both tests were used in this paper and were conducted under standard instructions. Table 2 provides basic information regarding the geo-engineering characteristics of the South Pars marls. These laboratory test results were used as a database for preparing the predictive model. In addition, the results of the model were verified by other methods.

### 3.3. Rock Material Database

**Figure 2:**
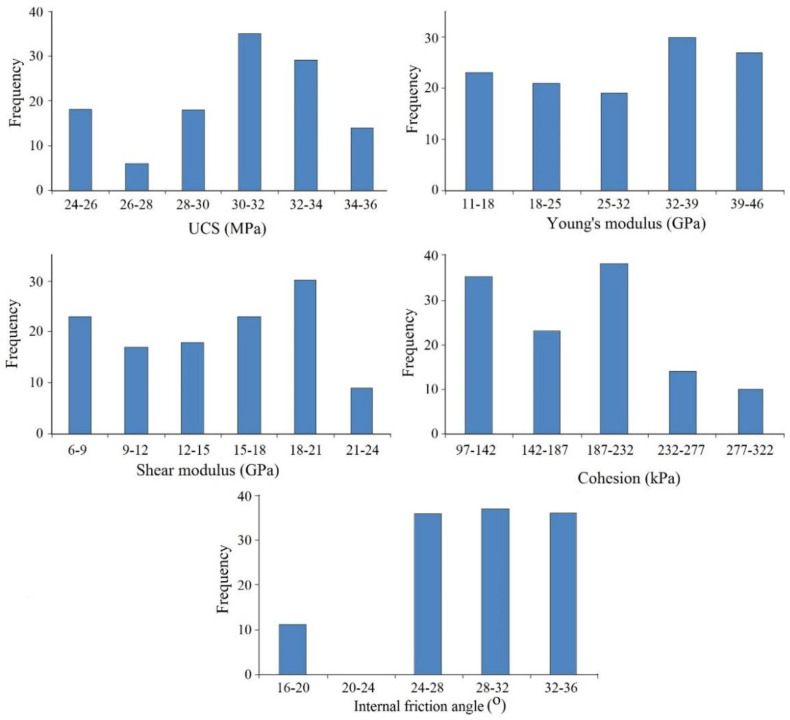
A histogram of the geomechanical indices.

Although there are various direct methods for estimating geomechanical indices, high implementation costs, which are time-consuming, along with problems related to sampling, sample preparation, and testing related to direct methods, have led to the option of utilizing indirect methods. The empirical relationships based on ordinary regression analysis have been used to develop experimental correlations by various researchers. However, since the information obtained in such a traditional procedure involves parameters such as the Coefficient of Determination (R^2^), which may not be proper for geotechnical projects involving sensitive materials, owing to the vast technological advancements in computer applications in geotechnics, researchers tend to use predictive models. As is known, a reliable predictive model requires a great deal of high-quality information about the target variables to produce results with high accuracy. The work presented in this study provides a comprehensive database of geomechanical indices (i.e., UCS, E, G, c, and φ) of the South Pars marlstones. A histogram of the variation of the geomechanical indices is illustrated in Figure 2.

**Table 1:** Sedimentary rock classification system by Pettijohn [48].

Sediment Group	Sedimentary Rock Classification	Percentage of Components (%)	
		Carbonate	Clay
Carbonate	Limestone	95–100	0–5
	Slightly argillaceous lime	85–95	5–15
	Argillaceous lime	75–85	15–25
Marl	Calcareous marl	65–75	25–35
	Marlstone	35–65	35–65
	Argillaceous marl	25–35	65–75
Clay	Calcareous mud	15–25	75–85
	Slightly argillaceous mud	5–15	85–95
	Mudstone	0–5	95–100

**Table 2:** The geo-engineering properties of the South Pars marls.

Parameter	Max	Min	Mean	Standard Dev.	Variance	Skewness
UCS (MPa)	34.72	24.17	29.44	2.982	8.895	−0.550
E (GPa)	45.30	11.70	28.50	10.17	103.5	−0.245
G (GPa)	22.10	6.00	14.05	5.152	25.45	−0.169
c (kPa)	320	97	208.5	59.52	354.2	0.221
φ (degree)	35	16	25.50	4.803	23.07	−0.794

## 4. Proposed Predictive Model

### 4.1. Deep Neural Network and Deep Learning

**Figure 3:**
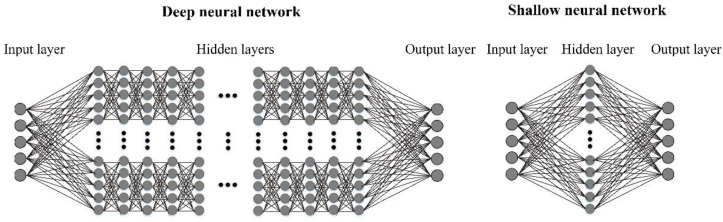
Topical shallow and deep neural network architectures [49].

Deep structured learning, or deep learning, is the main field of machine learning technology which is based on artificial neural networks with supervised, semi-supervised, or unsupervised learning approaches [49]. Deep learning techniques such as deep neural networks (DNN) have developed mathematically complex models to investigate multi-variable systems where they have produced results that are comparable to, and in some cases, surpass human expert performance. Artificial Neural Networks (ANNs) are in general inspired by the information processing and biological nervous system to provide an accurate understanding and predict outputs from input information. This capability of ANNs aids in providing predictive models and information processing for geomechanical key characteristics with high accuracy [50]. ANNs are mainly a collection of connected units or nodes called artificial neurons where each connection, named edges (like the biological synapses) can transmit signals and data to the other neurons. An artificial neuron that receives information and then processes it and then can signal neurons connected to it acts as a highly simplified human brain nerve system. Typically, these neurons are aggregated into layers which provide systematic learning by ANNs. The interconnection between these layers provides a powerful flexible tool for prediction and classification [49]. During learning, the mentioned interconnections are optimized and self-corrected based on error estimation by loss functions. In shallow learning, the layers are categorized as input, output, and middle (hidden) layers [50]. The concept of “deep” in deep learning refers to the utilization of multiple layers in the ANNs network. Since the application of shallow learning techniques such as perceptron can normally not be considered as highly accurate universal classifiers, more layers are required to provide detailed learned models. Deep learning considers a modified practical model to analyze the multiple variations with an unbounded number of layers for optimized classifications and predictions. A topical structure of shallow and deep neural networks is presented in Figure 3. As seen in this figure, the DNN networks consist of more hidden layers than shallow learning, which leads to providing more accurate results [49]. Hence, for these reasons, the presented article has utilized the DNN network to prepare the optimized predictive models. In general, the paper used a feed-forward deep neural network which is known as the DNN model. A deep neural network (DNN) is an artificial neural network (ANN) with multiple layers between the input and output layers. DNNs are feedforward networks in which data flows from the input layer to the output layer without looping back. At first, the DNN creates a map of virtual neurons and assigns random numerical values, or “weights”, to connections between them. The weights and inputs are multiplied and return an output between 0 and 1. If the network did not accurately recognize a particular pattern, an algorithm would adjust the weights. That way the algorithm can make certain parameters more influential until it determines the correct mathematical manipulation to fully process the data. Also, due to the variation of the input data, overfitting occurs when a model starts to memorize the training data instead of generalizing it to new data. Deep neural networks are prone to overfitting because they learn the different amount of parameters while building the model. A model having this many parameters can overfit the training data because it has sufficient capacity to do so. The “dropout” is the main function that is used in reducing the overfitting of data during the learning process. Dropout is a regularization method that approximates the training of a large number of neural networks with different architectures in parallel. Using dropout reduces the model overfitting significantly.

### 4.2. Data Acquisition

In order to provide information about rock material characteristics, a comprehensive site survey has been conducted which includes extensive sampling and testing. In this regard, even though geomechanical laboratory and in situ experiments are the most accurate method for gathering this information, it should be noted that performing such tasks lead to significant costs and time. As an alternative, empirical and/or predictive relationships can deliver a continuous dataset of rock material geomechanical properties as well. The important point of such indirect approaches is to provide appropriate results with less cost and time [49–51]. It should be noted that to obtain better convergence in the dataset, the data should be normalized, as illustrated by Equation (1) [26]:

(1) $$V_{\mathrm{norm}}=\frac{V_{t}-V_{\min}}{V_{\max}-V_{\min}}$$

where V_norm_ is the normalized value of the variable, V_t_ is the actual value estimated from experiments, and V_max_ and V_min_ are the maximum and minimum values of the dataset. As mentioned previously, the dataset was compiled using the 120 marlstone samples that were recovered from the South Pars region of Iran. As is well known, any predictive or correlational study must include independent (input) features or variables and a corresponding dependent (target) variable. In this study, the input data consists of the index geomechanical parameters of the intact marls that were recorded from the studied area in the training set which is tested in the testing set. The prediction results will be derived from the testing set prepared from the primary database. This database contains all sample results from the geotechnical laboratory.

### 4.3. Deep Neural Network Architecture

**Figure 4:**
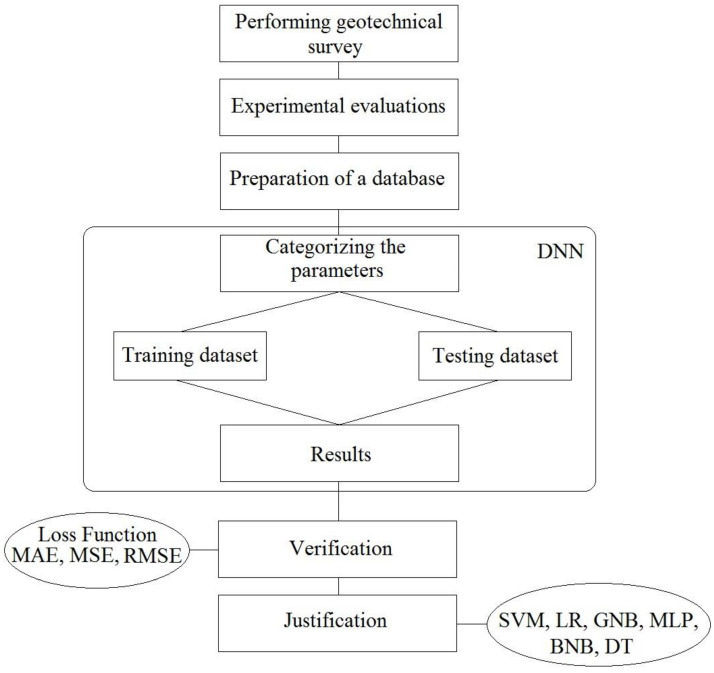
The implemented DNN model flowchart.

To demonstrate the application of the proposed DNN-based model, the gathered database which contained 120 data points pertaining to the testing and determination of the geomechanical characteristics formed the basic information set. The database was therefore divided into the training set and the test set. The training set contained 80% of the primary database, while the test set comprised the remaining 20%. The DNN algorithm was first applied to the training set before being evaluated on the test set. The evaluation procedure was implemented within a Python high-level programming language environment [49]. The structural flowchart of the DNN model is presented in Figure 4. After learning the procedure, the DNN-based model was controlled by the evaluation criterion and then tested on the test set.

### 4.4. Model Performance Studies

In machine learning and neural network classification, the confusion matrix (or error matrix) was used to control the accuracy, capability, and performance of the applied algorithms. The confusion matrix is a table layout that allows the visualization of the performance of the algorithm. Each row of the matrix represents instances from a predicted class, while each column represents instances from an actual class or vice versa [50]. This matrix has a positive impact on predictive analytics since it reports the number of true positives (an outcome where the model correctly predicts the positive class), true negatives (an outcome where the model correctly predicts the negative class), false positives (an outcome where the model incorrectly predicts the positive class), and false negatives (an outcome where the model incorrectly predicts the negative class). The confusion matrix estimates the detailed mere proportion of correct classifications as the accuracy of the predictive model for which accuracy yields misleading results if the trained-test data sets are unbalanced. In addition, this matrix can provide efficient information about precision and predictive model sensitivity and specificity, as illustrated in the following equations [49]:

(2) $$\mathrm{Precision}=\frac{\mathrm{True}\;\mathrm{Positives}\;}{\mathrm{True}\;\mathrm{Positives}\;+\;\mathrm{False}\;\mathrm{Positives}\;}$$

(3) $$\mathrm{Recall}/\mathrm{Sensitivity}=\frac{\mathrm{True}\;\mathrm{Positives}\;}{\mathrm{True}\;\mathrm{Positives}\;+\;\mathrm{False}\;\mathrm{Negatives}\;}$$

(4) $$\mathrm{Specificity}=\frac{\mathrm{True}\;\mathrm{Negatives}\;}{\mathrm{True}\;\mathrm{Negatives}\;+\;\mathrm{False}\;\mathrm{Positives}\;}$$

On the other hand, the F1-score, which is the harmonic mean of precision and recall, provides approximately the average of the two values when they are close, and is more generally the harmonic mean [49]:

(5) $$F1-\mathrm{score}=2\cdot \frac{\mathrm{Precision}\cdot \;\mathrm{Recall}}{\mathrm{Precision}+\mathrm{Recall}}$$

The overall accuracy represents the probability that an individual will be correctly classified by a test; that is, the sum of true positives plus true negatives divided by the total number of the individuals tested [49]:

(6) $$\mathrm{Accuracy}=\frac{\mathrm{True}\;\mathrm{Positives}\;+\;\mathrm{True}\;\mathrm{Negatives}\;}{\mathrm{True}\;\mathrm{Positives}\;+\;\mathrm{True}\;\mathrm{Negatives}\;+\;\mathrm{False}\;\mathrm{Positives}\;+\;\mathrm{False}\;\mathrm{Negatives}\;}$$

The other performance controllers in the predictive models are statistical error indices such as MAE, MSE, and RMSE. These relations are frequently used to measure the differences between the predicted (by a model) and the measured (by tests or observed) values [49]:

(7) $$\mathrm{MAE}=\frac{\sum_{t=1}^{n}\left|y_{i}-x_{i}\right|}{n}$$

(8) $$\mathrm{MSE}=\frac{1}{n}\overset{n}{\underset{i=1}{\sum }}{\left(y_{i}-x_{i}\right)}^{2}$$

(9) $$\mathrm{RMSE}=\sqrt{\frac{\sum_{t=1}^{n}{\left(y_{i}-x_{i}\right)}^{2}}{n}}$$

where *y_i_* is the predicted value and *x_i_* is the measured value. In the application of these indicators, the lower amount of computational error indicates better performance of the algorithms.

### 4.5. Model Verification

In order to justify the proposed model, the provided DNN-based predictive model was compared to several state-of-the-art benchmark machine learning techniques including the Support Vector Machine (SVM), Logistic Regression (LR), Gaussian Naïve Bayes (GNB), Multilayer Perceptron (MLP), Bernoulli Naïve Bayes (BNB) and Decision Tree (DT) classifiers. These classifiers are considered the most popular approaches to machine learning which are used to investigate the capability and accuracy of the proposed method in predictions. The methods were utilized as a comparative task to understand the performance of the DNN model as well as to estimate the statistical error indices along with the confusion matrix.

The mentioned classifiers are known as benchmark learning methods, which are considered as the most common machine learning procedures used by various researchers. SVM is a supervised network with associated learning algorithms that analyze data for classification and regression analysis. SVM uses statistical learning frameworks to predict the relations between the datasets and to extract the relevant features [50]. LR is a classifier that uses the probability of a certain class or event (such as pass/fail, win/lose, alive/dead, or healthy/sick) to determine classes between several objects by assigning a probability between 0 and 1. In fact, LR utilizes the logistic function to model binary dependent variables by using the log-odds (the logarithm of the odds) method [49]. GNB and BNB are two classifiers that are the variant of Naïve Bayes networks. Naïve Bayes is a group of supervised machine learning classification algorithms based on the Bayes theorem with strong (naïve) independence assumptions between the features [50]. MLP is a neural network-based supervised algorithm that is used to classify objects by using a feedforward network. MLP refers to networks composed of multiple layers of perceptrons, which can be categorized into at least three layers of nodes, concluded in an input layer, a hidden layer, and an output layer [49]. DT is a classifier that uses the decision support method (tree-like models) for decisions and their possible consequences. DT applies decision analysis to identify the most probable strategy to reach assessment goals [50]. These classifiers are the most common methods in machine learning to classify the various data sets and prepare predictive models, which are used as verifications for the DNN model. All predictive models use a comparative assessment (i.e., confusion matrix, evaluation criteria) and an error table (MAE, MSE, and RMSE).

## 5. Results and Discussion

**Figure 5:**
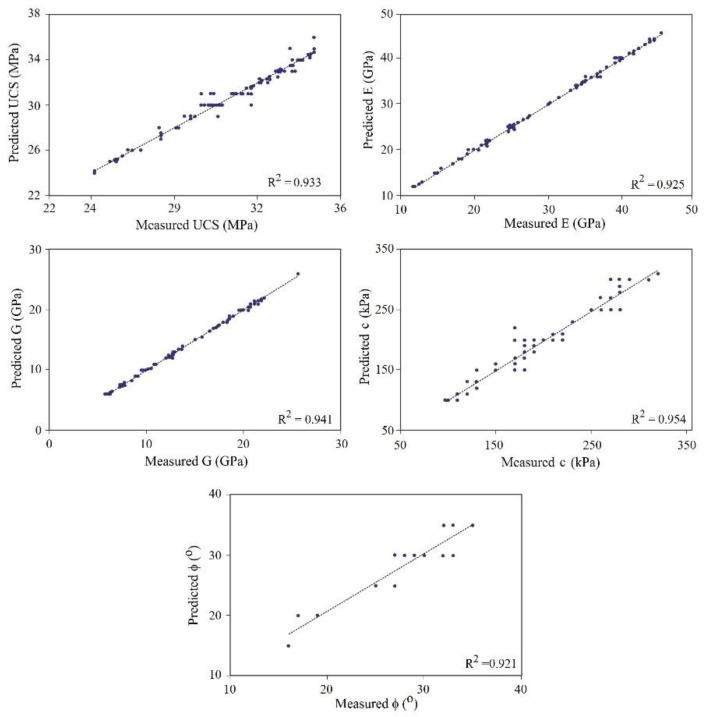
Correlation between the measured data and the predicted model for the geotechnical values.

**Figure 6:**
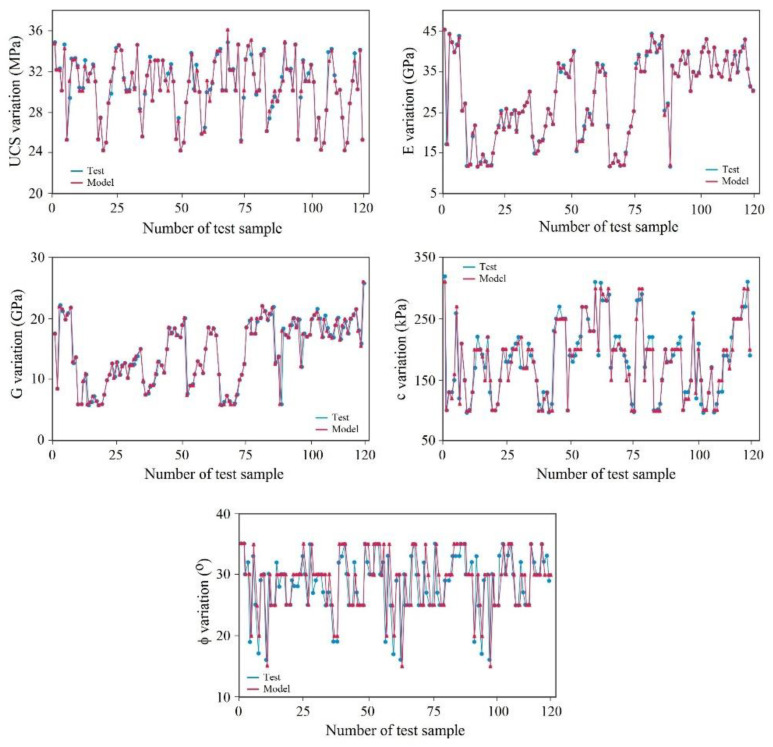
A comparison between the measured and predicted values based on the proposed method.

**Figure 7:**
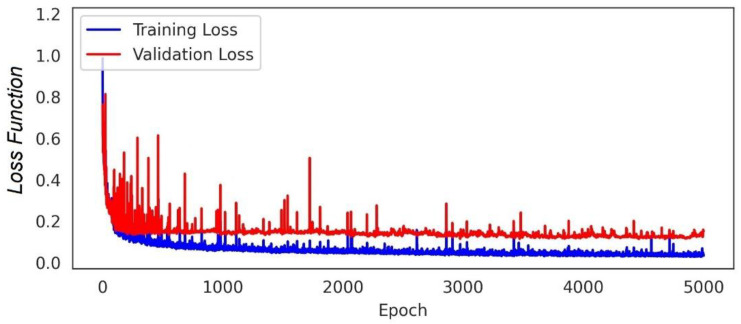
The loss function for the DNN model.

**Figure 8:**
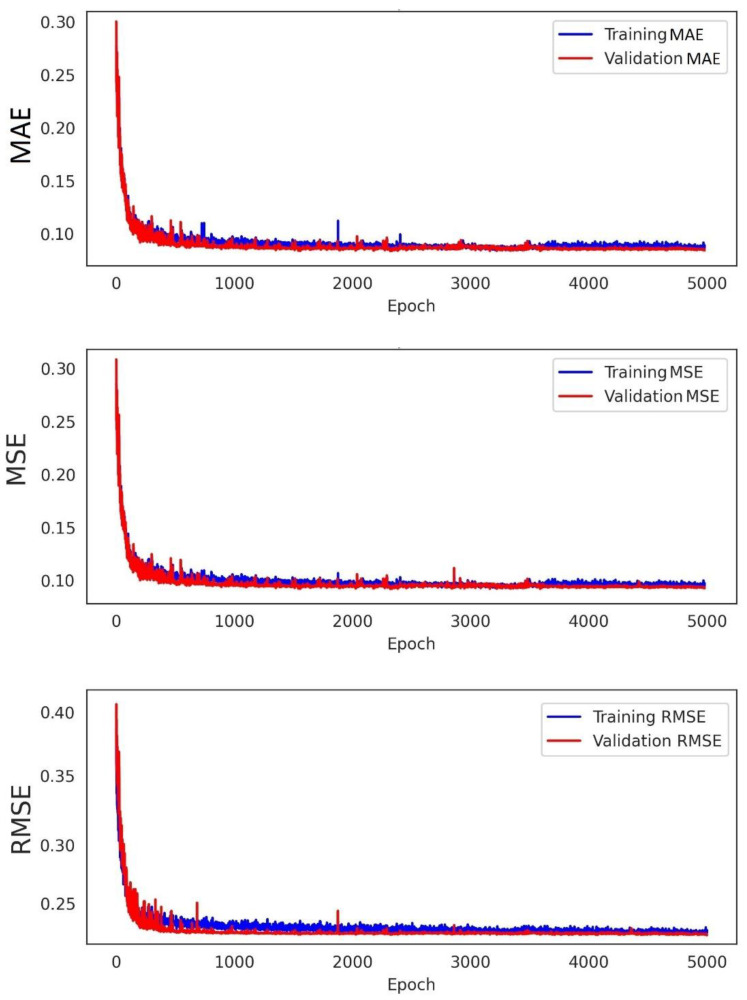
Prediction error evaluation for the DNN model and its components.

A database has been applied for the training and testing of the DNN network, which was divided into two subsets: the training set (80% of the main database) and the testing set (20% of the main database) both of which were implemented using the Python high-level programming language. The proposed deep learning model was verified based on the confusion matrix and the statistical error indices. In addition, it has been comparatively justified by using state-of-the-art benchmark machine learning techniques. The database was provided according to the comprehensive field survey, sampling, and testing performed in the South Pars region in southwest Iran. The obtained results are presented in Figure 5, Figure 6, Figure 7 and Figure 8. According to these figures, it can be stated that the DNN-based predictive model was confirmed to be a highly accurate tool for predicting rock material strength and stiffness properties. Referring to Figure 5, the predicted values of the geotechnical parameters are against the measured parameters. The measured parameters obtained from the laboratory geotechnical tests were performed in accordance with the procedure outlined by ASTM or ISRM. The results of the experiments pertain to the 120 marlstone samples that were recovered from different spatial locations of the South Pars region in southwest Iran. The samples were prepared and tested in the laboratory to estimate the geomechanical properties of the rock materials. The experimental results were used as comparative elements to assess the performance of the proposed model where the predicted values that were prepared by the DNN predictive model were compared with the measured values by using regression analysis. The coefficient of determination (R^2^) was used to estimate the variation of the dependent variable that was predictable from the independent variables. R^2^ represents the link between the observed outcomes and the observed predictor value, which normally ranges from 0 to 1, in that the closer it is to 1, the higher the accuracy of the data overlap. According to Figure 5, the estimated R2 for the geomechanical indices was 0.933 for UCS, 0.925 for E, 0.941 for G, 0.954 for c, and 0.921 for φ. Figure 6 compares the evaluated variations between the tested materials and those estimated by the model to provide a comparative perspective for evaluation. As demonstrated by this figure, the DNN model provides close results with the measured values.

The model was verified and justified by comparative procedures as seen in Figure 5 and Figure 6. The correlation coefficients and the error indices that were estimated for the models indicated that the proposed deep learning-based model has the highest accuracy (0.95) and precision (0.97) in evaluating the geomechanical properties. Table 3 provides information on the confusion matrix and the controlled learning of the applied models. According to the results, the proposed method has attained the closest predictive model to the real condition. In addition, by considering standard error evaluations, the proposed model has achieved less error than the other methods, with MAE = 0.13, MSE = 0.11, and RMSE = 0.17. This fact is provided in Figure 7 and Figure 8. Figure 7 illustrates the Loss Function for the training and test data sets of the model. Regarding this figure, the model errors have shown a significant reduction during the learning process (epochs). The process epochs reached 5000 and the error rate was reduced to less than 0.1 for the training and to less than 0.2 for the test process. The error function is commonly used to demonstrate the capability of the predictive model in producing information with low error. Figure 8 presents the error table of the DNN model regarding MAE, MSE, and RMSE. These indices represent the differences between the predicted values by a model and the measured values. According to the analysis, the estimated values for MAE, MSE, and RMSE are about 0.13, 0.11, and 0.17 for 5000 epochs, respectively. The reduction in the MAE, MSE, and RMSE values indicated the high accuracy of the prediction capacity of the model.

As mentioned in Table 3, which provides the performance assessment for verification of the DNN model by using various commend classifiers, the applied mode has achieved the highest accuracy (0.95) among other algorithms. The nearest algorithm to the DNN method belonged to the MLP (0.88) model. At a glance, it is evident that neural networks are superior to other classifiers in providing predictive information on rock properties. In general, the confusion matrix provides the point of view about predictive model performance as well as precision and accuracy indices. These factors aid the user in evaluating the capability of different models. Referring to the precision of the models utilized, the DNN model with the highest precision (0.97) is followed by the SVM model in second place (0.88) and the MLP model in third place (0.85).

**Figure 9:**
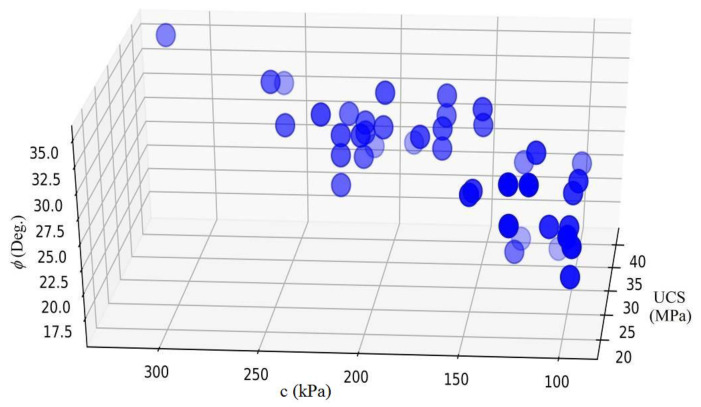
Variation chart for geotechnical parameters.

Regarding the results of the justification by the confusion matrix, the DNN model has provided accurate results for predicting the geomechanical properties of the marls. Thus, it can be comparatively used to cover the gaps in the estimation of the strength and stiffness parameters in South Pars. In this regard, a geomechanical characteristic and variation chart may be prepared by incorporating parallel studies that supply empirical results on marlstones of the region for a thorough geotechnical characterization of the marls in the South Pars region. Figure 9 illustrates the variation of USC, c, and φ in the South Pars region.

## 6. Conclusions

This article proposes a novel indirect predictive model to investigate the geomechanical properties and predict the strength characteristics of rock materials. The proposed method that incorporates a deep neural network to provide a comprehensive framework includes (1) establishing a database for the geomechanical properties and stiffness indices as determined by standard laboratory tests; (2) performing a deep learning procedure to predict the key factors of a rock material comprising marlstone; (3) verification of the predictive model by using the standard error indices and the confusion matrix; and (4) justification of the predictive model by using common benchmark machine learning classifiers such as SVM, LR, GNB, MLP, BNB, and DT. 

The benchmark classifiers are mainly different procedures that are used to predict the features from the main datasets. SVM uses supervised learning and linear regression to classify the data. LR uses the probability of a certain class or existing event such as pass/fail, win/lose, alive/dead, or healthy/sick to investigate the class of elements. Naïve Bayes applies the Bayes’ theorem with naïve to classify the features based on probabilistic assumptions. MLP is considered a feed-forward artificial neural network that uses multiple layers of perceptrons to classify the elements. DT is a decision support tool that uses a tree-like model (operations research technique) of decisions and their possible consequences. Although these models use different approaches in analyses, they are classified as machine learning procedures that help to verify the main algorithm regarding the performance and accuracy of the model.

The database was created by performing laboratory geotechnical tests on a total of 120 marlstone specimens recovered from the South Pars region in southwest Iran. According to the results of the performance analysis of the proposed model, the model showed good agreement with the measured data, which demonstrated the capability of the DNN method. In addition, based on the loss function, error matrix, and error indices, the proposed model achieved high accuracy (0.95) and precision (0.97) in evaluating the geomechanical parameters of the marlstone. By considering the Loss Function, the estimated error rate after 5000 epochs was reduced to 0.1 for the training dataset and to less than 0.2 for the test dataset, respectively. The predictive model has also provided the least error rate (MAE = 0.13, MSE = 0.11 and RMSE = 0.17) as compared to the other classifiers. This error table has indicated that the precision of the model is high in predicting geotechnical properties such as UCS, c, φ, E, and G. Estimation of the R^2^ for the predicted geomechanical parameters through the DNN model vs. those obtained with the experimental tests indicated that the predictive model had a high overlap with the test results. The estimated R^2^ was 0.933 for UCS, 0.925 for E, 0.941 for G, 0.954 for c, and 0.921 for φ.

## Figures and Tables

**Table 3:** The confusion matrix and the controlled learning models for the retrieved documents.

Classifier	Accuracy	Assessment score		
		Precision	Recall	F1-Score
SVM	0.71	0.88	0.71	0.71
LR	0.50	0.45	0.51	0.50
GNB	0.70	0.65	0.69	0.69
MLP	0.88	0.85	0.77	0.77
GNB	0.75	0.71	0.77	0.75
DT	0.45	0.65	0.65	0.40
DNN	0.95	0.97	0.95	0.95

Note: SVM: Support Vector Machine, LR: Logistic Regression, GNB: Gaussian Naïve Bayes, MLP: Multilayer Perceptron, BNB: Bernoulli Naïve Bayes, and DT: Decision Tree classifiers.

## Data Availability

The study did not report any data.
